# Whole genome sequence analysis of serum amino acid levels

**DOI:** 10.1186/s13059-016-1106-x

**Published:** 2016-11-24

**Authors:** Bing Yu, Paul S. de Vries, Ginger A. Metcalf, Zhe Wang, Elena V. Feofanova, Xiaoming Liu, Donna Marie Muzny, Lynne E. Wagenknecht, Richard A. Gibbs, Alanna C. Morrison, Eric Boerwinkle

**Affiliations:** 1 Human Genetics Center, University of Texas Health Science Center at Houston, Houston, TX USA; 2 Human Genome Sequencing Center, Baylor College of Medicine, Houston, TX USA; 3 Public Health Sciences, Wake Forest School of Medicine, Winston-Salem, NC USA

**Keywords:** Amino acids, Whole genome sequence, Metabolomics, Rare variants, Multi-ethnic

## Abstract

**Background:**

Blood levels of amino acids are important biomarkers of disease and are influenced by synthesis, protein degradation, and gene–environment interactions. Whole genome sequence analysis of amino acid levels may establish a paradigm for analyzing quantitative risk factors.

**Results:**

In a discovery cohort of 1872 African Americans and a replication cohort of 1552 European Americans we sequenced exons and whole genomes and measured serum levels of 70 amino acids. Rare and low-frequency variants (minor allele frequency ≤5%) were analyzed by three types of aggregating motifs defined by gene exons, regulatory regions, or genome-wide sliding windows. Common variants (minor allele frequency >5%) were analyzed individually. Over all four analysis strategies, 14 gene–amino acid associations were identified and replicated. The 14 loci accounted for an average of 1.8% of the variance in amino acid levels, which ranged from 0.4 to 9.7%. Among the identified locus–amino acid pairs, four are novel and six have been reported to underlie known Mendelian conditions. These results suggest that there may be substantial genetic effects on amino acid levels in the general population that may underlie inborn errors of metabolism. We also identify a predicted promoter variant in *AGA* (the gene that encodes aspartylglucosaminidase) that is significantly associated with asparagine levels, with an effect that is independent of any observed coding variants.

**Conclusions:**

These data provide insights into genetic influences on circulating amino acid levels by integrating -omic technologies in a multi-ethnic population. The results also help establish a paradigm for whole genome sequence analysis of quantitative traits.

**Electronic supplementary material:**

The online version of this article (doi:10.1186/s13059-016-1106-x) contains supplementary material, which is available to authorized users.

## Background

Conventional wisdom holds that common complex diseases are polygenic and rare Mendelian diseases are monogenic. Indeed the biology of human health and disease is complex and there is a continuum of genetic architectures. For example, ever since the seminal work of Goldstein and Brown with familial hypercholesterolemia [1], it is appreciated that a subset of individuals in the far tails of the phenotype distribution (e.g., LDL-cholesterol) may have a Mendelian form of a condition while others may have a polygenic predisposition. To gain a complete understanding of the genetic architecture of health and disease will require: 1) realization of the continuum of Mendelian and polygenic conditions; 2) consideration of the whole genome; and 3) multi-omic approaches that allow measurements of intermediate phenotypes closer to gene action and that bridge genome variation with inter-individual differences in disease risk.

Circulating blood levels of amino acids and whole genome sequence data combined with state-of-the-art annotation and analysis tools can help establish a paradigm for defining the genetic architecture of quantitative phenotypes. Rare recessive mutations in genes that lead to deficiencies or excess of specific amino acids are the root cause of a number of inborn errors of metabolism [2]. Inter-individual differences in several amino acids are risk factors for common disease (e.g., branched-chain and aromatic amino acids for diabetes) [3]. Amino acids are important components of protein metabolism and cell signaling. They reflect a variety of cellular and physiologic processes and may, therefore, mirror gene–environment interactions. Genome-wide association studies (GWAS) have identified common variants associated with multiple amino acid levels [4–6]. Low-frequency variants that modulate amino acid levels independent of known GWAS loci have also been reported using exome arrays and a targeted analytical approach for exome sequence data [7, 8]. To date, no study has assessed the impact of rare and low-frequency variations captured by systematic and comprehensive sequencing of the protein-encoding exons and whole genomes on amino acid levels in a multi-ethnic population. We used exon and whole genome sequencing in a sample of 3424 European and African Americans to investigate the genetic determinants of 70 blood amino acid levels. Significant effects discovered in African Americans (AA) were replicated in an independent set of European Americans (EA). This study demonstrates the utility of combining multi-omic data and the importance of intermediate phenotypes close to gene action for identifying regions of the genome influencing biologically and clinically relevant traits.

## Results

### Baseline characteristics

We sequenced exons and whole genomes and measured serum levels of 70 amino acids in 1872 AA for the discovery stage and 1552 EA for the replication stage among participants in the Atherosclerosis Risk in Communities (ARIC) study. Baseline characteristics of both the discovery and replication samples are shown in Additional file 1: Table S1. The mean age of the AA and EA participants was 52.7 and 54.7 years, respectively, and 65.2 and 54.9% of the samples were female. Prevalent diabetes was diagnosed in 16 and 8% of the AA and EA subjects, respectively, and 52 and 31%, respectively, had prevalent hypertension. In the AA samples, a total of 330,490 single nucleotide variants (SNVs) in the exons were captured by exome sequencing and 52,094,875 in the whole genomes; 94.8% of the SNVs were rare or low-frequency (minor allele frequency (MAF) ≤5%) in the exons and this number was 82.9% in the whole genomes. The proportion of variants within frequency bins characterized as rare (0% < MAF < 1%), low-frequency (1% ≤ MAF ≤ 5%), and common (MAF > 5%) is shown in Additional file 2: Figure S1.

**Fig. 1:**
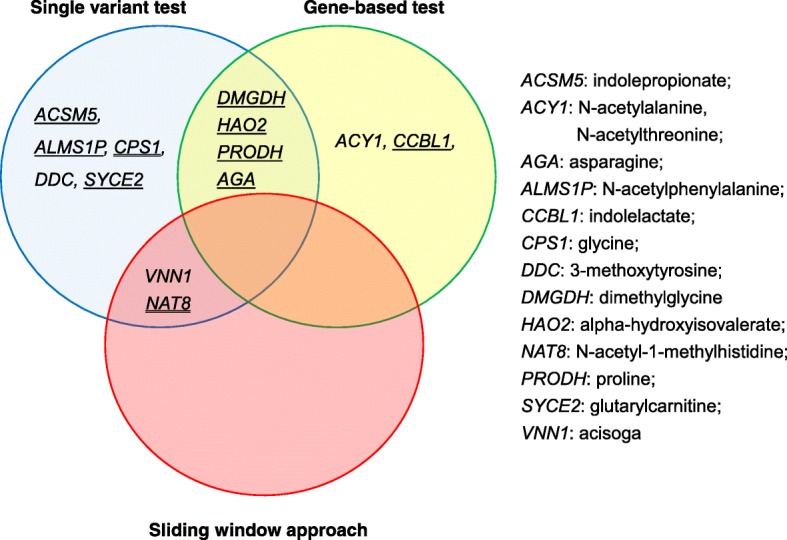
Identified significant genetic associations with serum amino acid levels. Gene names with a *single line* underneath indicate the association was reported in previous studies for European ancestry; gene names with *double lines* underneath indicate the association was reported in previous studies for both African and European ancestry. Gene names shown in the single variant test were assigned according to the leading common variant annotations

We used four approaches to examine the association of amino acid levels with genetic variants across the genome: 1) a gene exon approach; 2) an annotated regulatory motif approach; 3) a genome-wide sliding window approach; and 4) a single variant approach. The single variant approach analyzes the variants individually and the other three approaches collapse rare and low-frequency variants into a burden test because insufficient information is available for any one rare variant within a fixed sample size. The gene exon approach leverages the strength of the exome sequence data and the regulatory motif and sliding window approaches highlight the utility of whole genome sequence data. Each of these approaches is separately addressed in the following paragraphs. Overall, a total of 14 genetic loci–amino acid paired associations exceeded our a priori defined threshold for statistical significance in the discovery analysis in AA samples and were replicated in the EA samples. Within the 14 pairs, six loci–amino acid relationships were detected by more than one analytical approach (Fig. 1). Ten out of 14 pairs have been reported by previous GWAS, and the other four pairs are novel. A comparison between the 14 pairs and previous GWAS findings is provided in Additional file 1: Table S2.

### Gene exon approach

For the gene exon approach, we restricted our analysis to predicted functional variants with MAF ≤5%. A total of 15,589 genes with cumulative minor allele counts (cMAC) ≥7 were analyzed. We identified and replicated seven gene–amino acid pairs (*HAO2–*alpha-hydroxyisovalerate, *AGA–*asparagine, *DMGDH–*dimethylglycine, *CCBL1–*indolelactate, *ACY1–*N-acetylalanine and *ACY1*–N-acetylthreonine, *PRODH–*proline) with significant discovery *p* values (*P*_*dis*_) < 4.6 × 10^−8^ and a replication *p* value (*P*_*rep*_) <0.003 (Table 1). There were 12 to 30 rare and low-frequency variants involved within each of the identified genes. Detailed results for each rare and low-frequency variant involved in these genes are provided in Additional file 1: Table S3. A full list of identified gene–amino acid pairs regardless of successful replication is provided in Additional file 1: Table S4. Annotated functional variants in the six genes of the seven gene–amino acid pairs accounted for 0.6–3.6% of the variance in the amino acid levels, with the average being 1.8%. The six genes all encode enzymes, four of which directly catalyze reactions involving the identified amino acids as substrates or end products. The relationships between *AGA* and asparagine (*P*_*dis*_ = 1.3 × 10^−10^, *P*_*rep*_ = 2.7 × 10^−5^), dimethylglycine and *DMGDH* (*P*_*dis*_ = 3.2 × 10^−31^, *P*_*rep*_ = 8.1 × 10^−12^), N-acetylalanine, N-acetylthreonine and *ACY1* (*P*_*dis*_ = 4.1 × 10^−41^ and 1.1 × 10^−10^, *P*_*rep*_ = 3.9 × 10^−15^ and 4.7 × 10^−5^), proline and *PRODH* (*P*_*dis*_ = 1.4 × 10^−29^, *P*_*rep*_ = 1.5 × 10^−11^) are consistent with known autosomal recessive metabolic disorders. The gene exon results for the meta-analysis of the discovery and replication samples with *p* < 4.0 × 10^−6^ are provided in Additional file 1: Table S5.

**Table 1:** Gene exon-based results demonstrating a significant association among both discovery (*p* < 4.6 × 10^−8^) and replication (*p* < 0.003) stages for the T5 burden test

Metabolite	Gene	Discovery (AA)				Replication (EA)			
		*P*	Beta	cMAC	VarExp	*P*	Beta	cMAC	VarExp
Dimethylglycine	*DMGDH*	3.2 × 10^−31^	0.64	96	3.6%	8.1 × 10^−12^	0.39	73	1.7%
N-acetylthreonine	*ACY1*	1.1 × 10^−10^	0.12	239	0.6%	4.7 × 10^−5^	0.26	24	0.4%
N-acetylalanine	*ACY1*	4.1 × 10^−41^	0.16	239	1.5%	3.9 × 10^−15^	0.25	24	0.6%
Asparagine	*AGA*	1.1 × 10^−10^	0.34	157	1.4%	2.7 × 10^−5^	0.38	58	0.9%
Indolelactate	*CCBL1*	2.7 × 10^−21^	0.39	87	1.6%	1.1 × 10^−7^	0.26	33	0.5%
Alpha-hydroxyisovalerate	*HAO2*	1.6 × 10^−8^	0.64	21	0.8%	8.2 × 10^−6^	0.41	18	0.5%
Proline	*PRODH*	1.4 × 10^−29^	0.14	324	1.4%	1.5 × 10^−11^	0.09	295	0.7%

*cMAC* cumulative minor allele count, *VarExp* variance explained by the loci

### Regulatory motif approach

Defining regulatory motifs away from protein-encoding genes is a major activity of modern genome sciences. Projects such as ENCODE [9] and GTEx [10] are defining noncoding regions of the genome that have important biologic function, including regulation of gene expression. We analyzed a total of 21,040 annotated regulatory motifs with cMAC ≥7 across the genome, and statistical significance was defined as *P*_*dis*_ < 3.4 × 10^−8^. Although two regulatory motifs exceeded our a priori significance threshold for discovery in the AA samples, they did not replicate in the EA samples (Additional file 1: Table S6). To help up-weight predicted functional variants, the regulatory motif analysis was repeated and weighted by the combined annotation dependent depletion (CADD) scores [11], but the results did not change substantially from those of the unweighted analyses (Additional file 2: Figure S2). The regulatory motif results for the meta-analysis of the discovery and replication samples with *p* <4.0 × 10^−6^ are provided in Additional file 1: Table S7.

### Sliding window approach

We next applied a sliding window approach to analyze rare and low-frequency variation (MAF ≤5%) aggregated by 4-kb windows with a 2-kb skip length using burden tests to scan the entire genome. A total of 1,337,499 windows (668,748 non-overlapping windows) with cMAC ≥7 were analyzed. We identified and replicated two genomic regions influencing two amino acid levels (*P*_*dis*_ < 1.1 × 10^−9^ and *P*_*rep*_ < 0.01; Table 2). One is a 130-kb region at 2p13.2, where two windows in the region were associated with N-acetyl-1-methylhistidine levels (lowest window *P*_*dis*_ = 1.6 × 10^−15^, *P*_*rep*_ = 3.9 × 10^−4^). *ALMS1* and *NAT8*, two neighboring genes residing in this 130-kb region, have been previously reported to be related to N-acetyl amino acids levels [4, 6]. The other region is located at 6q23.2 where a single window 46 kb downstream of *VNN1* was associated with acisoga. Detailed results for each rare and low-frequency variant involved in the identified windows are provided in Additional file 1: Table S3. A full list of identified significant sliding window–amino acid pairs regardless of successful replication is provided in Additional file 1: Table S8. The sliding window results for the meta-analysis of the discovery and replication samples with *p* < 4.0 × 10^−6^ are provided in Additional file 1: Table S9.

**Table 2:** Sliding windows demonstrating a significant association among both discovery (*p* < 1.1 × 10^−9^) and replication (*p* < 0.01) stages for the T5 burden test

Metabolite	Discovery (AA)					Replication (EA)				
	Window (gene)	*P*	Beta	cMAC	VarExp	Window (gene)	*P*	Beta	cMAC	VarExp
N-acetyl-1-methylhistidine	Chr2: 73744005–73748004 (*NAT8*)	1.6 × 10^−15^	0.12	933	1.0%	Chr2: 73744005–73748004 (*NAT8*)	0.0004	0.17	156	0.5%
N-acetyl-1-methylhistidine	Chr2: 73614005–73618004 (*NAT8*)	6.2 × 10^−11^	−0.11	728	0.7%	Chr2: 73614005–73618004 (*NAT8*)	0.005	−0.07	336	0.2%
Acisoga	Chr6: 132952009–132956008 (*VNN1*)	9.4 × 10^−10^	0.06	1504	0.4%	Chr6: 132952009–132956008 (*VNN1*)	0.009	−0.04	764	0.1%

*cMAC* cumulative minor allele count, *VarExp* variance explained by the loci

### Single variant approach

In addition to rare and low-frequency variants, we conducted a survey of the genome investigating common SNVs with MAF >5%. Eleven single variant–amino acid associations reached the significance threshold at both the discovery and replication stages (*P*_*dis*_ < 7.1 × 10^−10^ and *P*_*rep*_ < 0.003; Table 3). These 11 common variants accounted for 0.7–9.7% of the variance of amino acids levels, with an average of 2.3%. The 11 SNVs all resided in protein-encoding gene regions, six of which encode enzymes that catalyze the reaction of the corresponding metabolite as a substrate or product. Among the significant findings, two gene–amino acid associations are novel (3-methoxytyrosine and *DDC*, and acisoga and *VNN1*) and there are two loci, *DDC* and *CPS1*, in which mutations are known to cause autosomal recessive metabolic disorders. A full list of identified significant single variant–amino acid pairs regardless of successful replication is provided in Additional file 1: Table S10. The single variant results for the meta-analysis of the discovery and replication samples with *p* < 5.0 × 10^−8^ are provided in Additional file 1: Table S11.

**Table 3:** Single variant results demonstrating a significant association among both discovery (*p* < 7.1 × 10^−10^) and replication (*p* < 0.003) stages

Metabolite	Variant information					Discovery (AA)				Replication (EA)			
	Gene	SNP	Function	Chr:position	REF/ALT	MAF	Beta	*P*	Var Exp	MAF	Beta	*P*	Var Exp
Glycine	*CPS1*	rs1047891	Missense	2:211540507	C/A	0.37	0.09	4.5 × 10^−19^	1.3%	0.31	0.16	4.9 × 10^−45^	3.6%
Dimethylglycine	*DMGDH*	rs933683	Intronic	5:78324003	G/T	0.44	−0.15	2.3 × 10^−14^	1.9%	0.29	−0.09	9.5 × 10^−6^	0.7%
Asparagine	*AGA*	rs11131799	Intronic	4:178363378	G/A	0.49	−0.14	2.4 × 10^−10^	2.5%	0.36	−0.26	3.9 × 10^−23^	4.5%
N-acetyl-1-methylhistidine	*NAT8*	rs13538	Missense	2:73868328	A/G	0.48	0.34	3.3 × 10^−75^	9.7%	0.23	0.51	3.4 × 10^−85^	14.2%
Glutarylcarnitine	*SYCE2*	rs8012	Missense	19:13010520	A/G	0.19	−0.12	9.5 × 10^−14^	1.2%	0.46	−0.11	2.5 × 10^−17^	1.5%
N-acetyl phenylalanine	*ALMS1P*	rs13431529	Intronic	2:73876041	G/C	0.49	0.09	4.3 × 10^−10^	1.0%	0.23	0.06	1.2 × 10^−5^	0.4%
3-Methoxytyrosine	*DDC*	rs11575302	Silent	7:50607694	G/A	0.15	0.15	2.5 × 10^−17^	1.5%	0.02	0.19	1.4 × 10^−7^	0.5%
Indolepropionate	*ACSM5*	rs8044331	Intronic	16:20450302	T/C	0.42	−0.17	5.3 × 10^−10^	1.8%	0.22	−0.11	0.001	0.5%
Alpha-hydroxyisovalerate	*HAO2*	rs17023507	UTR5	1:119923247	C/T	0.10	−0.25	1.6 × 10^−13^	1.9%	0.002	−0.64	0.001	0.4%
Proline	*PRODH*	rs1814288	Intronic	22:18923383	C/T	0.30	−0.06	7.8 × 10^−12^	0.7%	0.21	−0.03	0.003	0.1%
Acisoga	*VNN1*	rs2272996	Missense	6: 133015271	T/C	0.19	0.18	8.1 × 10^−16^	0.2%	0.27	0.26	4.8 × 10^−34^	5.1%

*REF/ALT* reference allele and alternative allele, *MAF* minor allele frequency, *VarExp* variance explained by the loci

### Conditional analyses

Across all analytic approaches, six of the region–amino acid associations have been reported in previous GWAS: *AGA*–asparagine, *DMGDH*–dimethylglycine, *HAO2*–alpha-hydroxyisovalerate, *PRODH*–proline, *CCBL1*–idnolelactate, and two sliding windows close to *NAT8* with N-acetyl-1-methylhistidine. We performed conditional analyses in order to examine whether sequencing data were able to identify independent region-based effects at loci highlighted by previous GWAS. Results of the region-based conditional analyses are shown in Table 4. Low-frequency variants in *AGA*, *DMGDH*, *HAO2*, *PRODH*, and *CCBL1* were associated with amino acid levels independent of the known GWAS lead variants. The association of low-frequency variants in the two sliding windows near *NAT8*, however, was strongly attenuated after adjusting for rs13538, the lead variant identified by previous GWAS. Among these six associations, we examined whether any GWAS findings can be explained by rare and low-frequency variants. In one case, rs248386, the significance of the lead variant identified by previous GWAS of dimethylglycine levels was largely diminished after conditioning on the burden of rare and low-frequency variants in *DMGDH* (Additional file 1: Table S12). We next performed conditional analyses to determine whether the lead single common variants for nine locus–amino acid associations were independent from the lead variants identified by GWAS. In three of these cases (rs13538–*NAT8*, rs1047891–*CPS1*, and rs8012–*SYCE2*), we identified the same lead variant as previous GWAS. The remaining lead variants we discovered in AA samples (rs11131799–*AGA*, rs933683–*DMGDH*, rs1814288–*PRODH*, rs13431529–*ALMS1P*, rs8044331–*ACSM5*, and rs17023507–*HAO2*) were generally independent of those identified by previous GWAS (Additional file 1: Table S13).

**Table 4:** Conditional analysis of selected regions adjusting for the lead common variant identified by previous genome-wide association studies

Metabolite	Region	Type	GWAS Lead SNV	Discovery (AA)		Replication (EA)	
				*P*_*unadjusted*_	*P*_*adjusted*_	*P*_*unadjusted*_	*P*_*adjusted*_
Indolelactate*	*CCBL1*	Gene	rs15676	1.3 × 10^−20^	1.1 × 10^−20^	2.1 × 10^−6^	4.1 × 10^−6^
N-acetyl-1-methylhistidine	Chr2: 73744005–73748004 (*NAT8*)	Window	rs13538	1.6 × 10^−15^	0.005	4.0 × 10^−4^	0.2
N-acetyl-1-methylhistidine	Chr2: 73614005–73618004 (*NAT8*)	Window	rs13538	6.2 × 10^−11^	0.9	0.005	0.8
Asparagine*	*AGA*	Gene	rs4690522	6.8 × 10^−10^	9.1 × 10^−10^	1.5 × 10^−5^	6.0 × 10^−8^
Dimethlyglycine*	*DMGDH*	Gene	rs248386	1.1 × 10^−26^	4.3 × 10^−27^	4.4 × 10^−11^	4.5 × 10^−10^
Alpha-hydroxyisovalerate*	*HAO2*	Gene	rs12141041	1.5 × 10^−5^	3.0 × 10^−5^	9.3 × 10^−5^	2.0 × 10^−4^
Proline*	*PRODH*	Gene	rs2540641	1.4 × 10^−26^	1.7 × 10^−26^	1.3 × 10^−12^	1.2 × 10^−13^

*Unadjusted results may differ from main analysis because only individuals with both exome sequencing and whole genome sequencing were included in the conditional analysis. *SNV* single nucleotide variant

## Discussion

We identified and replicated 14 associations between genetic loci and serum amino acid levels, all in or neighboring genes encoding enzymes. Four of the associated gene–amino acid pairs were novel (*DDC–*3-methoxytyrosine, *VNN1–*acisoga, *ACY1–*N-acetylalanine, and *ACY1–*N-acetylthreonine). Six of the loci–amino acid associations were identified by more than one analytical approach. In most cases, rare and low-frequency variants in the regions identified in this study were associated with amino acids independent of common variants previously identified by GWAS. Six of the gene–amino acid pairs identified here are known to underlie Mendelian disorders. Notably, among the four analytical approaches proposed in this study, analyses focusing on regulatory motifs was the only setting where there was no significant and replicated amino acid associations.

Amino acids are the building blocks of proteins. Humans can synthesize 11 of the 20 standard amino acids and the remaining nine essential amino acids must be obtained from dietary sources. The genetic loci identified in this study are all associated with non-essential amino acids or amino acid derivatives, although previous GWAS have reported multiple common variants that are associated with levels of nine essential amino acids [6, 12–14]. Given the nature of amino acid biosynthesis and the properties of the enzyme-encoding genes, it is of note that six of the identified enzymes directly catalyze reactions involving the amino acid as a substrate or end product.

Understanding the genetic bases of inherited metabolic disease has been a focus of human genetics for a long time. In this study, we identified six genes (*DMGDH*, *AGA*, *ACY1*, *PRODH*, *DDC*, *CPS1*) that have been previously implicated in recessive metabolic disorders, four of which show direct relationships to the amino acids identified here: mutations in *AGA* are known to cause aspartylglucosaminuria (MIM 208400); mutations in *DMGDH* cause dimethylglycine dehydrogenase deficiency (MIM 605850); mutations in *ACY1* cause aminoacylase-1 deficiency (MIM 609924); and mutations in *PRODH* are known to cause hyperprolinemia type I (MIM 239500). Although the other two loci did not directly affect the identified amino acid levels, there is evidence suggesting that the two genes play a role in their regulation. *DDC* participates in tyrosine metabolism (DBGET: R02080) and mutations in it are known to causearomatic L-amino acid decarboxylase deficiency (AADC; MIM 608643). The identified amino acid 3-methoxytyrosine is one of the main biochemical markers of AADC [15]. *CPS1* (carbamoyl phosphate synthetase I) encodes an ammonia ligase (DBGET: R00149) and deficiency of the CPS1 protein (MIM 608307) leads to hyperammonemia. Glycine is a precursor of ammonia (DBGET: R01221) and, as such, accumulates in the liver and kidneys under the condition of excess ammonia [16]. *DMGDH*–dimethylglycine, *AGA*–asparagine, *PRODH*–proline, and *CPS1*–glycine associations were reported by several previous studies (Additional file 1: Table S2), while the *ACY1*–N-acetylthreonine/N-acetylalanine and *DDC*–3-methoxytyrosine associations are novel. Our findings support that genetic variation impacts inter-individual differences in amino acid levels in the general population in addition to causing recessive inborn errors of metabolism.

The data reported here provide new insight into the genes influencing blood amino acid levels. For example, *CCBL1*, which encodes kynurenine aminotransferase 1, was associated with three lactate derivatives, including indolelactate, phenyllactate (PLA), and 3-(4-hydroxyphenyl)lactate. Kynurenine aminotransferase 1 is known to be involved in tryptophan metabolism (DBGET: T01001, hsa00380), where it converts kynurenine, an intermediate of the tryptophan degradation pathway, into kynurenic acid [17], a neurotoxic compound associated with schizophrenia [18]. One of the three amino acids, indolelactate, is also part of tryptophan metabolism (DBGET: hsa00380). A common variant in *CCBL1* has been reported to be related to indolelactate in populations of European ancestry [13], and we observed that rare and low-frequency variants in *CCBL1* were associated with indolelactate in both AA and EA samples independent of the reported common variant. Because of the neurotoxic effect of kynurenic acid, inhibition of the kynurenine pathway is a therapeutic strategy for neurodegenerative disease [19, 20]. Current available drugs are indoleamine-pyrrole 2,3-dioxygenase (IDO) inhibitors, which inhibit the conversion of tryptophan to kynurenine. We identified rare and low-frequency variants in *IDO1*, encoding IDO, associated with low levels of kynurenine, suggesting that participants carrying functional mutations in *IDO1* may show neuroprotection. Phenylalanine, tyrosine, and tryptophan have common steps in their biosynthesis pathway (DBGET:map00400). Interestingly, besides tryptophan metabolism, the other two identified lactate derivatives, PLA and 3-(4-hydroxyphenyl)lactate, are involved in phenylalanine and tyrosine metabolism. Both PLA and 3-(4-hydroxyphenyl)lactate are elevated in phenylketonuria and hyperphenylalaninemia [21], which if untreated may result in mental impairment and other neurologic disorders (MIM 261600 and 261640). Our results indicate that rare and low-frequency variants in *CCBL1* are associated with increased levels for all three lactate derivatives. Future studies are warranted to dissect the mechanism of the observed associations and the possibility of *CCBL1* as a novel drug target for neurologic disorders.

The results reported here generate new hypotheses that future studies can investigate. One example is the association between a common missense variant in *VNN1* and acisoga. Acisoga is a newly described amino acid involved in polyamine metabolism. Although polyamines are ubiquitous small molecules, acisoga is the only polyamine measured in our metabolomics panel. *VNN1* encodes vanin 1, which shares extensive sequence similarity with biotinidase. The function for *VNN1* is not well studied; however, it possesses pantetheinase activity, which may play a role in oxidative-stress response [22]. There is convincing evidence that altered polyamine metabolism is involved in many diseases, and drugs altering polyamine levels therefore may have a variety of important disease targets [23]. The results presented here provide preliminary directions for further research on polyamine metabolism and the *VNN1* gene.

The analysis strategy and results presented here establish a paradigm for whole genome sequence analysis of quantitative risk factor phenotypes. There is compelling evidence based on GWAS that common variants confer relatively small increments in risk and explain only a small proportion of the heritability [24]. Assessment of rare and low-frequency variants, specifically non-coding rare and low-frequency variants, in relation to human health is largely incomplete. Whole genome sequencing data offer an opportunity to characterize rare and low-frequency variations and variations outside of the usual protein-encoding regions. The UK10K and GoT2D projects [25, 26] have demonstrated success identifying novel findings utilizing whole genome sequencing, but this success has been limited compared to GWAS, in part due to the limited statistical power. Compared to studies of complex diseases, the study of quantitative phenotypes, such as amino acid levels which are proximal to gene function, can dramatically maximize statistical power. Our study successfully identified and replicated four novel findings, demonstrating the feasibility of analyzing whole genome sequences in the context of intermediate quantitative phenotypes to promote novel biologically relevant findings.

Although the majority of the findings in our study reside in coding regions, we were able to identify non-coding loci that contribute to amino acid levels. For example, a common intronic variant, rs11131799, was shown to be associated with asparagine levels, independent of coding variants in *AGA* (*AGA*, *P*_*unadjusted*_ = 1.1 × 10^−10^, *P*_*adjusted*_ = 2.4 × 10^−9^). Conditioning on *AGA* coding variants did not markedly alter the non-coding locus association. *AGA* encodes the enzyme aspartylglucosaminidase, which breaks down glycoproteins by hydrolyzing N-acetylglucosamine–asparagine linkages, thereby releasing asparagine. Rs11131799, annotated as a predicted promoter variant, is highly associated with *AGA* expression levels (http://genenetwork.nl/biosqtlbrowser/). Some of the variants involved in the 4-kb window are annotated as predicted deleterious by CADD [11] and FATHMM-MKL [27]. A previous study identified an association between asparagine and the *ASPG* locus, encoding asparaginase [13], which catalyzes the hydrolysis of asparagine to aspartic acid. Interestingly, our lead variant for the *AGA*–asparagine association (rs11131799) occurred in both AA and EA participants, while the previously reported lead variant (rs4690522) was only observed in EA participants. The two variants were in strong linkage disequilibrium in EA participants, but not in linkage disequilibrium in AA participants, suggesting that rs4690522 may have simply been a proxy for rs11131799 in previous studies. The data reported here suggest that blood asparagine levels may be influenced not only by the coding regions but also by some regulatory elements. Further annotation information is warranted to dissect the two non-coding regions in relation to asparagine levels.

Among the four analytical approaches proposed in this study, the analysis of regulatory motifs was the only approach that did not yield novel findings. If we consider effect sizes seen in the other analysis approaches, these results reemphasize that improvements in annotation, particularly non-coding regulatory elements, are necessary. It is likely that the high density of non-functional variants in the hypothesized regulatory motifs overwhelms the sparser functional variants included in a burden test. Alternatively, single rare and low-frequency variants with large effects may be scarce in annotated regulatory elements of the human genome.

Strengths of this study include the use of direct sequencing, as opposed to genotyping and imputation. By using sequencing data, we were able to interrogate low-frequency, rare, and private variants that are not covered by genotyping and imputation. Even for variants accessible by both approaches, sequencing avoids the measurement error generated by imputation, which can be large for rare variants. The advantages of sequencing are particularly important for fine-mapping, since differences in imputation quality among variants can obstruct the search for the most likely causal variant. An additional strength of this study is the joint calling of variants in a larger pooled sample of studies conducted in the same laboratory, including ARIC. By increasing the sample size during the calling of variants, the ability to correctly call rare variants is enhanced [28].

The discovery sample for this study was AA, a population with a high level of genetic diversity, to promote novel findings. Also, AA are relatively under-represented in large-scale genomics research. To our knowledge, there is no AA sample for which both whole genome sequencing and multi-amino acid measurements are available to perform replication. Therefore, EA were used as the replication sample. Our focus here is the similar associations detected in both AA and EA. For the associations that were not replicated in EA, population-specific genetic variation and effects are possible reasons in addition to the original observation being a type I error. The variants included in aggregate tests differed between our discovery (AA) and replication (EA) samples due to ancestry-specific variants as well as allele frequency differences among shared variants. The variance explained by a genetic locus provides an estimate about the proportion of phenotypic variation that is attributed to inter-individual differences in DNA sequence. In this study, the variance explaining amino acid levels ranges from 0.4 to 9.7% among AA. Our previous GWAS reported 5 to 20% variance explaining differing levels of five amino acids [6], and the range of variance explaining differences in amino acid levels varied among Caucasians, such as 1–10% [29] or 1–25% [13]. To our knowledge, there is no trans-ethnic genetic association study of amino acid levels. Nevertheless, our exploratory trans-ethnic meta-analysis provided insights for future studies. Further investigation is warranted to evaluate these and additional findings in multiple ethnic groups.

## Conclusions

By integrating -omic technologies into deeply phenotyped populations, we show that sequencing variants affect the levels of multiple human amino acids among two ethnicities. These data and results identify new avenues of gene function, novel molecular mechanisms, and potentially diagnostic targets for multiple diseases.

## Methods

### Study population and metabolome measurements

The Atherosclerosis Risk in Communities (ARIC) study is a prospective epidemiological study designed to investigate the etiology and predictors of cardiovascular disease. It enrolled 15,792 individuals aged 45–64 years from four US communities (Forsyth County, NC; Jackson, MS; suburbs of Minneapolis, MN; and Washington County, MD) in 1987–89 (baseline) and followed them for four completed visits in 1990–92, 1993–95, 1996–98, and 2011–13. A detailed description of the ARIC study design and methods is published elsewhere [30]. Amino acid levels were measured using fasting serum samples collected at the baseline examination in 1987–1989 among ARIC selected AA and EA. A total of 89 amino acids were detected and semi-quantified by Metabolon Inc. (Durham, USA) using an untargeted, gas chromatography–mass spectrometry and liquid chromatography–mass spectrometry (GC-MS and LC-MS)-based metabolomic quantification protocol (Additional file 2: Supplemental methods) [31, 32]. Amino acids were excluded if: 1) more than 25% of the samples had values below the detection limit; or 2) the Pearson correlation coefficients between 2010 and 2014 measurements were <0.3 (Additional file 2: Supplemental methods). After this assessment, 70 metabolites were included in the present study.

### Exome sequencing

Isolated DNA from AA and EA for exon sequencing were further processed using the Baylor College of Medicine Human Genome Sequencing Center (BCM-HGSC) VCRome 2.1 reagent (42 Mb, NimbleGen) [33], and all samples were paired-end sequenced using Illumina GAII or HiSeq instruments. Details about sequencing, variant calling, and variant quality control are provided in Additional file 2: Supplemental methods. Variants were annotated using ANNOVAR [34] and dbNSFP v2.0 [35] according to the reference genome GRCh37 and National Center for Biotechnology Information RefSeq.

### Whole genome sequencing

Whole genome sequencing data for AA and EA were generated at BCM-HGSC using Nano or PCR-free DNA libraries and the Hiseq 2000 instrument (Illumina, Inc., San Diego, CA, USA). Methods for the whole genome sequencing of the ARIC study samples were described elsewhere [36]. Briefly, individuals were sequenced at sevenfold average depth on Illumina HiSeq instruments and variant calling was completed using goSNAP (https://sourceforge.net/p/gosnap/git/ci/master/tree/). Details about sequencing, variant calling, and variant quality control are provided in Additional file 2: Supplemental methods. Whole genome sequencing variants were annotated across regions and functional domains using the Whole Genome Sequencing Annotation (WGSA) pipeline [37]. The 3′ and 5′ UTRs of a gene were determined using ANNOVAR [34] annotations based on the RefSeq gene model [38]. The promoter of a gene was defined based on the overlap between the permissive set of CAGE peaks reported by the FANTOM5 project [39] and the 5-kb upstream region determined by the ANNOVAR annotation based on the RefSeq gene model. The enhancers and the target genes of the enhancers were defined based on the permissive set of enhancers and enhancer–promoter pairs reported by the FANTOM5 project. In the case of an undesignated enhancer–gene pair, we assigned an enhancer to the nearest gene.

### Statistical analyses

Metabolomic data points lying outside the 1^st^–99^th^ percentile of each amino acid level were winsorized among each measurement respectively. Levels below the detectable limit of the assay were imputed with the lowest detected value for that amino acid in all samples. Amino acid levels were then natural log-transformed prior to the analyses.

Because our primary focus was on rare and low-frequency variants, we aggregated rare and low-frequency variants (MAF ≤5%) in groups based on gene exons, regulatory motifs, or sliding windows. Gene-based aggregation tests are designed for rare and low-frequency coding variants. The analytical unit is an annotated gene. All annotated coding variants, such as splicing, stop-gain, stop-loss, nonsynonymous, and indels within the gene were aggregated for the analysis. The regulatory motifs included annotated enhancers, the 3′ and 5′ UTRs, and promoter of a gene. The sliding window approach is designed to aggregate rare and low-frequency variants according to their physical position regardless of annotated function. Based on our previous experience [36], sliding windows were defined as 4 kb in length and began at position 0 bp for each chromosome, with a skip length of 2 kb. Within each annotated unit, a burden test (T5) [40] was used, adjusting for age, sex, and the first three principal components (PCs). We further adjusted for estimated glomerular filtration rate (eGFR) [41], an indicator of kidney function, since multiple amino acid levels were associated with eGFR [42]. The T5 burden test collapses variants with MAF ≤5% into a single genetic score to evaluate the joint effects of rare and low-frequency alleles. We also conducted single variant analysis for all individual variants with MAF >5% using an additive genetic model with the same adjustments. For each approach, the variance explained (VarExp) was calculated using the effect allele frequency (*p*) and beta (*β*) from the analyses and the variance of the quantitative trait (*σ* ^*2*^) using the formula VarExp = *β* ^*2*^/*σ* ^*2*^ × 2 × *p* × (1 − *p*) [43]. In addition, we also applied the CADD scores [11] as variant weights to the regulatory motifs. The weights were defined as the difference between raw CADD scores and the minimum CADD score scaled by the range of the raw CADD scores and were introduced into the T5 burden test using its quartic form. The analytical models were the same as described above. All analyses were carried out using the R seqMeta package [44].

The significance threshold for the gene-based analysis is defined as *P*_*dis*_ < 4.6 × 10^−8^ for the discovery stage adjusting for 15,589 genes and 70 amino acids and *P*_*rep*_ < 0.003 for the replication stage adjusting for 15 significant gene–amino acid pairs identified in the discovery stage. The significance threshold for the regulatory motifs analysis is defined as *P*_*dis*_ < 3.4 × 10^−8^ for the discovery stage adjusting for 21,040 genes and 70 amino acids. The significance threshold for the sliding window approach is defined as *P*_*dis*_ < 1.1 × 10^−9^ for the discovery stage adjusting for 668,748 non-overlapping windows and 70 amino acids and *P*_*rep*_ < 0.01 for the replication stage adjusting for five significant window–amino acid pairs identified in the discovery stage. The significance threshold for the single variant analysis is defined as *P*_*dis*_ < 7.1 × 10^−10^ for the discovery stage adjusting for one million independent common variants [45] and 70 amino acids and *P*_*rep*_ < 0.003 for the replication stage adjusting for 16 significant single variant–amino acid pairs identified in the discovery stage. We consider an association novel if it has not been reported in previous GWAS or candidate gene study. We also performed trans-ethnic meta-analysis among the discovery and replication samples to provide additional insight into the genetic loci discovery.

Regions associated with amino acid levels using the gene-based or sliding window approaches that have already been identified by previous GWAS were selected for inclusion in the conditional analyses. We reexamined each of the selected associations, additionally adjusting the region-based association for the lead common variant identified by the GWAS, and vice versa. To adjust the GWAS variants for the identified regions, we computed the T5 burden and used it as a covariate. We also performed a conditional analysis for our single variant findings when these overlapped with regions identified by GWAS, adjusting our lead single variant for the lead variant identified by GWAS and vice versa.

## Additional files

Additional file 1: Tables S1–S13. (XLSX 195 kb)
Additional file 2: Supplemental methods and **Figures S1–S2**. (PDF 215 kb)

## References

[1] Brown MS, Goldstein JL (1986). A receptor-mediated pathway for cholesterol homeostasis. Science.

[2] Scriver C, Beaudet A, Sly W, Valle D, Childs B, Kinzler K, Vogelstein B. The Metabolic and Molecular Bases of Inherited Disease. 8th edn: New York City:McGraw-Hill Companies, Inc.; 2000.

[3] Wang TJ, Larson MG, Vasan RS, Cheng S, Rhee EP, McCabe E, Lewis GD, Fox CS, Jacques PF, Fernandez C (2011). Metabolite profiles and the risk of developing diabetes. Nat Med.

[4] Suhre K, Shin SY, Petersen AK, Mohney RP, Meredith D, Wagele B, Altmaier E (2011). CardioGram, Deloukas P, Erdmann J, et al. Human metabolic individuality in biomedical and pharmaceutical research. Nature.

[5] Rhee EP, Ho JE, Chen MH, Shen D, Cheng S, Larson MG, Ghorbani A, Shi X, Helenius IT, O’Donnell CJ (2013). A genome-wide association study of the human metabolome in a community-based cohort. Cell Metab.

[6] Yu B, Zheng Y, Alexander D, Morrison AC, Coresh J, Boerwinkle E (2014). Genetic determinants influencing human serum metabolome among African Americans. PLoS Genet.

[7] Yu B, Li AH, Muzny D, Veeraraghavan N, de Vries PS, Bis JC, Musani SK, Alexander D, Morrison AC, Franco OH (2015). Association of rare loss-of-function alleles in HAL, serum histidine: levels and incident coronary heart disease. Circ Cardiovasc Genet.

[8] Rhee EP, Yang Q, Yu B, Liu X, Cheng S, Deik A, Pierce KA, Bullock K, Ho JE, Levy D (2016). An exome array study of the plasma metabolome. Nat Commun.

[9] Encode Project Consortium (2012). An integrated encyclopedia of DNA elements in the human genome. Nature.

[10] GTEx Consortium (2013). The Genotype-Tissue Expression (GTEx) project. Nat Genet.

[11] Kircher M, Witten DM, Jain P, O’Roak BJ, Cooper GM, Shendure J (2014). A general framework for estimating the relative pathogenicity of human genetic variants. Nat Genet.

[12] Williams SR, Yang Q, Chen F, Liu X, Keene KL, Jacques P, Chen WM, Weinstein G, Hsu FC, Beiser A (2014). Genome-wide meta-analysis of homocysteine and methionine metabolism identifies five one carbon metabolism loci and a novel association of ALDH1L1 with ischemic stroke. PLoS Genet.

[13] Shin SY, Fauman EB, Petersen AK, Krumsiek J, Santos R, Huang J, Arnold M, Erte I, Forgetta V, Yang TP (2014). An atlas of genetic influences on human blood metabolites. Nat Genet.

[14] Raffler J, Friedrich N, Arnold M, Kacprowski T, Rueedi R, Altmaier E, Bergmann S, Budde K, Gieger C, Homuth G (2015). Genome-wide association study with targeted and non-targeted NMR metabolomics identifies 15 novel loci of urinary human metabolic individuality. PLoS Genet.

[15] Hyland K, Surtees RA, Rodeck C, Clayton PT (1992). Aromatic L-amino acid decarboxylase deficiency: clinical features, diagnosis, and treatment of a new inborn error of neurotransmitter amine synthesis. Neurology.

[16] van de Poll MC, Soeters PB, Deutz NE, Fearon KC, Dejong CH (2004). Renal metabolism of amino acids: its role in interorgan amino acid exchange. Am J Clin Nutr.

[17] Passera E, Campanini B, Rossi F, Casazza V, Rizzi M, Pellicciari R, Mozzarelli A (2011). Human kynurenine aminotransferase II--reactivity with substrates and inhibitors. FEBS J.

[18] Linderholm KR, Skogh E, Olsson SK, Dahl ML, Holtze M, Engberg G, Samuelsson M, Erhardt S (2012). Increased levels of kynurenine and kynurenic acid in the CSF of patients with schizophrenia. Schizophr Bull.

[19] Stone TW, Forrest CM, Darlington LG (2012). Kynurenine pathway inhibition as a therapeutic strategy for neuroprotection. FEBS J.

[20] Chen Y, Guillemin GJ (2009). Kynurenine pathway metabolites in humans: disease and healthy States. Int J Tryptophan Res.

[21] Spaapen LJ, Ketting D, Wadman SK, Bruinvis L, Duran M (1987). Urinary D-4-hydroxyphenyllactate, D-phenyllactate and D-2-hydroxyisocaproate, abnormalities of bacterial origin. J Inherit Metab Dis.

[22] Zhang B, Lo C, Shen L, Sood R, Jones C, Cusmano-Ozog K, Park-Snyder S, Wong W, Jeng M, Cowan T (2011). The role of vanin-1 and oxidative stress-related pathways in distinguishing acute and chronic pediatric ITP. Blood.

[23] Pegg AE (2009). Mammalian polyamine metabolism and function. IUBMB Life.

[24] Manolio TA, Collins FS, Cox NJ, Goldstein DB, Hindorff LA, Hunter DJ, McCarthy MI, Ramos EM, Cardon LR, Chakravarti A (2009). Finding the missing heritability of complex diseases. Nature.

[25] Fuchsberger C, Flannick J, Teslovich TM, Mahajan A, Agarwala V, Gaulton KJ, Ma C, Fontanillas P, Moutsianas L, McCarthy DJ, et al. The genetic architecture of type 2 diabetes. Nature. 2016;536:41–7. 10.1038/nature18642 PMC5034897 27398621

[26] Consortium UK, Walter K, Min JL, Huang J, Crooks L, Memari Y, McCarthy S, Perry JR, Xu C, Futema M (2015). The UK10K project identifies rare variants in health and disease. Nature.

[27] Shihab HA, Rogers MF, Gough J, Mort M, Cooper DN, Day IN, Gaunt TR, Campbell C (2015). An integrative approach to predicting the functional effects of non-coding and coding sequence variation. Bioinformatics.

[28] Grove ML, Yu B, Cochran BJ, Haritunians T, Bis JC, Taylor KD, Hansen M, Borecki IB, Cupples LA, Fornage M (2013). Best practices and joint calling of the HumanExome BeadChip: the CHARGE Consortium. PLoS One.

[29] Kettunen J, Tukiainen T, Sarin AP, Ortega-Alonso A, Tikkanen E, Lyytikainen LP, Kangas AJ, Soininen P, Wurtz P, Silander K (2012). Genome-wide association study identifies multiple loci influencing human serum metabolite levels. Nat Genet.

[30] The ARIC investigators (1989). The Atherosclerosis Risk in Communities (ARIC) Study: design and objectives. Am J Epidemiol.

[31] Ohta T, Masutomi N, Tsutsui N, Sakairi T, Mitchell M, Milburn MV, Ryals JA, Beebe KD, Guo L (2009). Untargeted metabolomic profiling as an evaluative tool of fenofibrate-induced toxicology in Fischer 344 male rats. Toxicol Pathol.

[32] Evans AM, DeHaven CD, Barrett T, Mitchell M, Milgram E (2009). Integrated, nontargeted ultrahigh performance liquid chromatography/electrospray ionization tandem mass spectrometry platform for the identification and relative quantification of the small-molecule complement of biological systems. Anal Chem.

[33] Bainbridge MN, Wang M, Wu Y, Newsham I, Muzny DM, Jefferies JL, Albert TJ, Burgess DL, Gibbs RA (2011). Targeted enrichment beyond the consensus coding DNA sequence exome reveals exons with higher variant densities. Genome Biol.

[34] Wang K, Li M, Hakonarson H (2010). ANNOVAR: functional annotation of genetic variants from high-throughput sequencing data. Nucleic Acids Res.

[35] Liu X, Jian X, Boerwinkle E (2013). dbNSFP v2.0: a database of human non-synonymous SNVs and their functional predictions and annotations. Hum Mutat.

[36] Morrison AC, Voorman A, Johnson AD, Liu X, Yu J, Li A, Muzny D, Yu F, Rice K, Zhu C (2013). Whole-genome sequence-based analysis of high-density lipoprotein cholesterol. Nat Genet.

[37] Liu X, White S, Peng B, Johnson AD, Brody JA, Li AH, Huang Z, Carroll A, Wei P, Gibbs R (2016). WGSA: an annotation pipeline for human genome sequencing studies. J Med Genet.

[38] O’Leary NA, Wright MW, Brister JR, Ciufo S, Haddad D, McVeigh R, Rajput B, Robbertse B, Smith-White B, Ako-Adjei D (2016). Reference sequence (RefSeq) database at NCBI: current status, taxonomic expansion, and functional annotation. Nucleic Acids Res.

[39] Forrest AR, Kawaji H, Rehli M, Baillie JK, de Hoon MJ, Haberle V, Lassmann T, Consortium F, the RP, Clst (2014). A promoter-level mammalian expression atlas. Nature.

[40] Li B, Leal SM (2008). Methods for detecting associations with rare variants for common diseases: application to analysis of sequence data. Am J Hum Genet.

[41] Levey AS, Stevens LA, Schmid CH, Zhang YL, Castro AF, Feldman HI, Kusek JW, Eggers P, Van Lente F, Greene T (2009). A new equation to estimate glomerular filtration rate. Ann Intern Med.

[42] Yu B, Zheng Y, Nettleton JA, Alexander D, Coresh J, Boerwinkle E (2014). Serum metabolomic profiling and incident CKD among African Americans. Clin J Am Soc Nephrol.

[43] Locke AE, Kahali B, Berndt SI, Justice AE, Pers TH, Day FR, Powell C, Vedantam S, Buchkovich ML, Yang J (2015). Genetic studies of body mass index yield new insights for obesity biology. Nature.

[44] seqMeta R package. http://cran.r-project.org/web/packages/seqMeta/index.html.

[45] Pe’er I, Yelensky R, Altshuler D, Daly MJ (2008). Estimation of the multiple testing burden for genomewide association studies of nearly all common variants. Genet Epidemiol.

[46] Rich SS, Wang ZY, Sturcke A, Ziyabari L, Feolo M, O’Donnell CJ, Rice K, Bis JC, Psaty BM (2016). Rapid evaluation of phenotypes, SNPs and results through the dbGaP CHARGE Summary Results site. Nat Genet.

