# Supplementary material for: Whole genome sequence analysis of serum amino acid levels
Source: Genome Biol. 2016 Nov 24;17:237. doi: 10.1186/s13059-016-1106-x (PMC5123402; doi:10.1186/s13059-016-1106-x)

SUPPLEMENTAL MATERIAL

Supplemental Methods

Amino Acids Measurements and Batch Effects Correction

The amino acids are measured at Metabolon Inc. (Durham, NC), using untargeted gas chromatography-mass spectrometry and liquid chromatography-mass spectrometry (GC/LC-MS) based metabolomic quantification protocols [1, 2]. Serum samples are extracted and prepared using Metabolon's standard solvent extraction method. The extracted samples are split into equal parts for analysis on the GC/MS and LC/MS platforms. Instrument variability is determined by calculating the median relative standard deviation (SD) for the internal standards that are added to each sample prior to injection into the mass spectrometers. Overall process variability are determined by calculating the median relative SD for all endogenous metabolites (i.e. non-instrument standards) present in 100% of the technical replicate samples. Since the measurements span multiple days, a data normalization step are performed to correct variation resulted from instrument inter-day tuning differences. The amino acids are identified by comparison to an in-house generated authentic standard library that includes retention time, molecular weight, preferred adducts, in-source fragments, and associated fragmentation spectra of the intact parent ion. For this study, a total of 89 amino acids were detected and semi-quantified, and 74 out of 89 amino acids had missing values ≤ 25%. We designed a set of 97 samples to measure their amino acids using baseline serum samples at both 2010 and 2014. We calculated the Pearson correlation coefficients (r^2) between the 97 pairs for these 74 amino acids, and mild batch effect was observed. Among 74 amino acids, the r^2 was ranged from 0.09 to 0.98, with mean at 0.70 and median at 0.74. We also compared the correlation between creatinine measured at baseline by the Jaffé method with those measured recently by LC-MS. Results show reasonable correlation for r2 equals 0.89. We excluded 4 amino acids with poor correlation coefficient ($r^2 < 0.3$) for analysis, therefore, 70 amino acids were examined in this study.

Exome Sequencing Variant Calling and Quality Control

The DNA samples were constructed into Illumina paired-end pre-capture libraries according to the manufacturer's protocol. The complete protocol and oligonucleotide sequences are accessible from the Baylor College of Medicine Human Genome Sequencing Center (BCM-HGSC) website (https://www.hgsc.bcm.edu/content/protocols-sequencing-library-construction). Two, four or six pre-capture libraries were pooled together and then hybridized to Nimblegen exome capture array (HGSC VCRome 2.1 design [3] (42Mb, NimbleGen) and sequenced in paired-end mode in a single lane on the Illumina HiSeq 2000 platform. Illumina sequence analysis was performed using the HGSC Mercury analysis pipeline (https://www.hgsc.bcm.edu/content/mercury). Pooled samples were de-multiplexed using the Consensus assessment of sequence and variation (CASAVA) software. Reads were then mapped to the Genome Reference Consortium Human Build 37 (GRCh37) human reference sequence using Burrows-Wheeler Aligner (BWA) [4] producing Binary Alignment/Map (BAM) files. Aligned reads were then recalibrated using the Genome Analysis Toolkit (GATK) [5] along with BAM sorting, duplicate read marking, and realignment near insertions or deletions (indels).

The Atlas2 [6] suite was used to call variants and produce high-quality variant call files (VCF) [7]. Each single nucleotide variant (SNV) call was filtered based on the following criteria to produce a high-quality variant list: low SNV posterior probability (<0.95), low variant read count (<3), variant read ratio <0.25 or >0.75, strand-bias of more than 99% variant reads in a single strand direction, or total coverage less than 10-fold. All variant calls filtered by these criteria, and reference calls with less than 10-fold coverage, were set to missing. The variant call filters were the same for indels except a total coverage less than 30-fold was used. Variant-level quality control steps excluded variants outside the exon capture regions (VChrome 2.1), multi-allelic sites, missing rate >20%, and mean depth of coverage >500-fold. Variants not meeting Hardy-Weinberg equilibrium expectations in ancestry-specific groups ($P<5\times10^{-6}$) were also excluded. Sample-level quality control metrics were calculated by cohort and ancestry group. A sample was excluded for missingness >20%, or if compared to the other samples it fell less than 6 standard deviations (SD) for mean depth, more than 6 SD for singleton count, or outside of 6 SD for heterozygote to homozygote ratio or a transition-to-transversion (Ti/Tv) ratio.

Whole Genome Sequencing Variant Calling and Quality Control

Whole genome sequence (WGS) data was generated by BCM-HGSC. DNA samples were constructed into Illumina paired-end libraries according to the manufacturer's protocol (Illumina Multiplexing_SamplePrep_Guide_1005361_D) and sequenced on the Hiseq 2000 (Illumina, Inc.; San Diego, CA) in a pooled format to generate a minimum of 18 unique aligned Gbp per sample. Methods for WGS of ARIC study participants have been described in detail in Morrison et al [8]. Individuals of African and European ancestry were sequenced at 7.4-fold average depth on Illumina HiSeq instruments and variant calling was completed using goSNAP [9], which employed GATK, SNPTools and GotCloud as callers, each in joint calling mode, and took an ensemble consensus approach to generate the high quality variant call set. The per sample genotyping and reference panel independent imputation and phasing were done using SNPTools. Across 5,297 samples from the Cohorts for Health and Aging Research in Genomic Epidemiology (CHARGE) Consortium, 72.8 million SNV were called with a Ti/Tv ratio of 2.13. A total of 59.7% of the SNVs are novel compared to dbSNP v142 as most of the novel variants are rare due to the large sample size. Compared to a subset of CHARGE samples with whole exome sequencing (n=4,612), the sensitivity and specificity of the WGS call set is 63.6% and 99.9%, respectively, and compared to an overlapping set of single nucleotide polymorphism (SNP) array data (n=3,533), the false discovery rate (FDR) is 1.6%. Variant-level quality assurance was achieved by excluding variants with site level inbreeding coefficient <-0.9. Variants not meeting Hardy-Weinberg equilibrium exact test expectations in ancestry-specific groups (p-value $<1\times10^{-14}$) were also excluded. Sample-level quality control and quality assurance checks included principal component analysis (PCA) to identify possible population substructure and sample abnormalities. The set of variants for PCA was restricted to variants with minor allele frequency (MAF) >5% and linkage disequilibrium between variants of $r^2<0.30$. A total of 40 ARIC AA individuals identified as outliers by PCA were removed from further analyses. Higher-order principal components showed minor levels of population structure. After sample-level quality control, a total of 1,860 AA and 1,705 EA from the ARIC study were available for the genotype-phenotype analyses.

Reference

1. Evans AM, DeHaven CD, Barrett T, Mitchell M, Milgram E: **Integrated, nontargeted ultrahigh performance liquid chromatography/electrospray ionization tandem mass spectrometry platform for the identification and relative quantification of the small-molecule complement of biological systems.** *Anal Chem* 2009, **81:**6656-6667.
2. Ohta T, Masutomi N, Tsutsui N, Sakairi T, Mitchell M, Milburn MV, Ryals JA, Beebe KD, Guo L: **Untargeted metabolomic profiling as an evaluative tool of fenofibrate-induced toxicology in Fischer 344 male rats.** *Toxicol Pathol* 2009, **37:**521-535.
3. Bainbridge MN, Wang M, Wu Y, Newsham I, Muzny DM, Jefferies JL, Albert TJ, Burgess DL, Gibbs RA: **Targeted enrichment beyond the consensus coding DNA sequence exome reveals exons with higher variant densities.** *Genome Biol* 2011, **12:**R68.
4. Li H, Durbin R: **Fast and accurate short read alignment with Burrows-Wheeler transform.** *Bioinformatics* 2009, **25:**1754-1760.
5. DePristo MA, Banks E, Poplin R, Garimella KV, Maguire JR, Hartl C, Philippakis AA, del Angel G, Rivas MA, Hanna M, et al: **A framework for variation discovery and genotyping using next-generation DNA sequencing data.** *Nat Genet* 2011, **43:**491-498.
6. Challis D, Yu J, Evani US, Jackson AR, Paithankar S, Coarfa C, Milosavljevic A, Gibbs RA, Yu F: **An integrative variant analysis suite for whole exome next-generation sequencing data.** *BMC Bioinformatics* 2012, **13:**8.
7. Danecek P, Auton A, Abecasis G, Albers CA, Banks E, DePristo MA, Handsaker RE, Lunter G, Marth GT, Sherry ST, et al: **The variant call format and VCFtools.** *Bioinformatics* 2011, **27:**2156-2158.
8. Morrison AC, Voorman A, Johnson AD, Liu X, Yu J, Li A, Muzny D, Yu F, Rice K, Zhu C, et al: **Whole-genome sequence-based analysis of high-density lipoprotein cholesterol.** *Nat Genet* 2013, **45:**899-901.
9. https://sourceforge.net/p/gosnap/git/ci/master/tree/.

Supplemental Figure

Figure S1. Proportion of Single Nucleotide Variants within frequency bins in African Americans: a) Whole Genome Sequencing and b) Whole Exome Sequencing

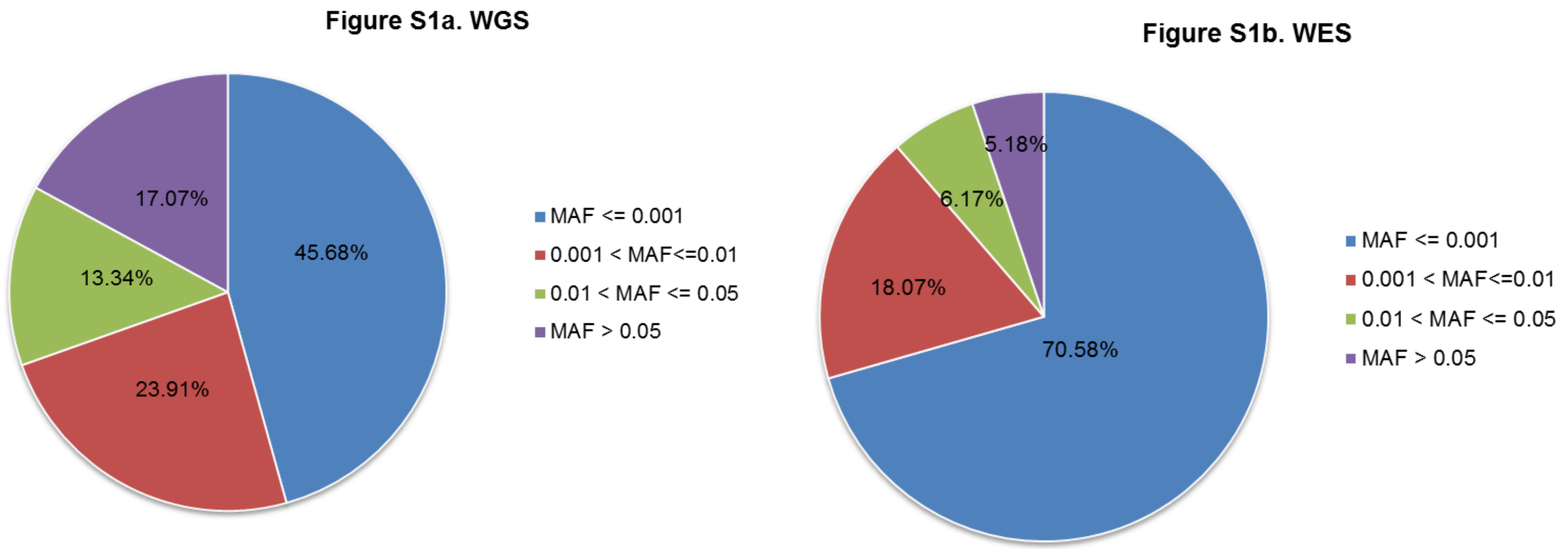

Figure S2. QQ-plot for unweighted tests involving regulatory motifs compared to weighted tests incorporating quartic-scaled CADD scores for two significant loci identified in the discovery samples: a) N-acetylalanine and b) N-acetyl-1-methylhistidine.

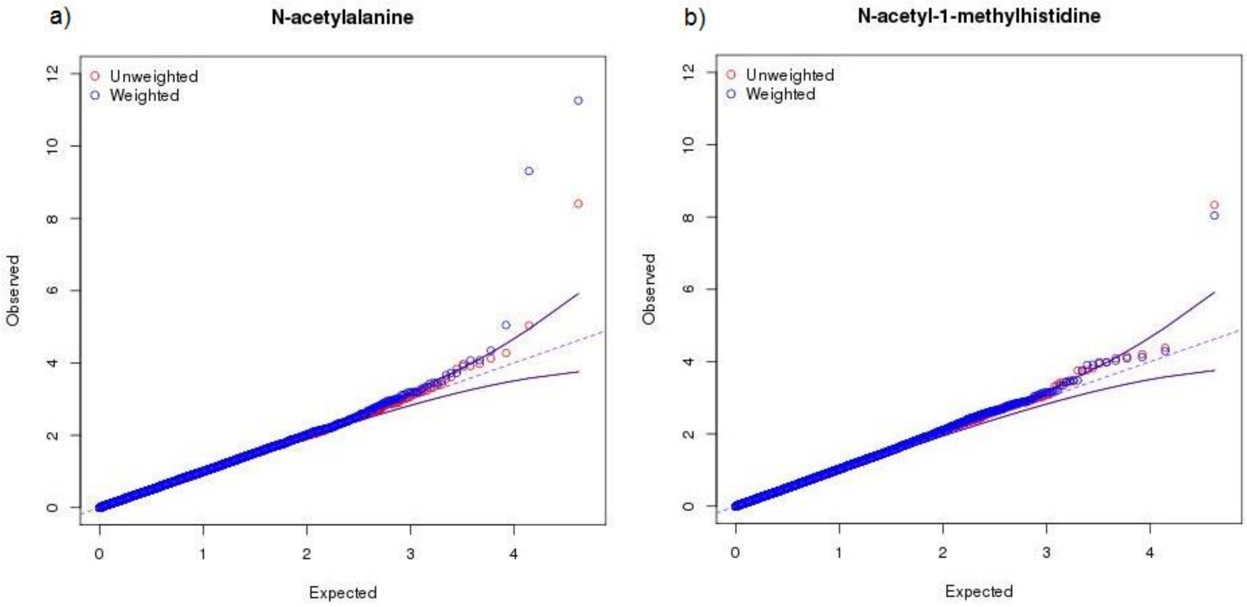

Supplement: Additional file 2: — Supplemental methods and Figures S1–S2. (PDF 215 kb) [file 13059_2016_1106_MOESM2_ESM.pdf]
